# *GCKR* Variants Increase Triglycerides While Protecting from Insulin Resistance in Chinese Children

**DOI:** 10.1371/journal.pone.0055350

**Published:** 2013-01-31

**Authors:** Yue Shen, Lijun Wu, Bo Xi, Xin Liu, Xiaoyuan Zhao, Hong Cheng, Dongqing Hou, Xingyu Wang, Jie Mi

**Affiliations:** 1 Graduate School of Peking Union Medicine College, Beijing, People’s Republic of China; 2 Department of Epidemiology, Capital Institute of Pediatrics, Beijing, People’s Republic of China; 3 Institute of Maternal and Child Health Care, School of Public Health, Shandong University, Shandong, People’s Republic of China; 4 Laboratory of Human Genetics, Beijing Hypertension League Institute, Beijing, People’s Republic of China; Pennington Biomedical Research Center, United States of America

## Abstract

**Background:**

Variants in gene encoding glucokinase regulator protein (*GCKR*) were found to have converse effects on triglycerides and glucose metabolic traits. We aimed to investigate the influence of *GCKR* variants for triglycerides and glucose metabolic traits in Chinese children and adults.

**Methods and Results:**

We genotyped two *GCKR* variants rs1260326 and rs1260333 in children and adults, and analyzed the association between two variants and triglycerides, glucose, insulin and HOMA-IR using linear regression model, and estimated the effect on insulin resistance using logistic regression model. Rs1260326 and rs1260333 associated with increased triglycerides in children and adults (*p*<0.05). In children, both variants significantly reduced insulin (*p*<0.05. for rs1260326, *β* = −0.07; for rs1260333, *β* = −0.07) and HOMA-IR (*p*<0.05. for rs1260326, *β* = −0.03; for rs1260333, *β* = −0.03). There were significant associations between two variants and insulin resistance for children. Under co-dominant model, for CT vs. CC, OR is 0.83 (95%CI 0.69–1.00) for rs1260326, and 0.83 (95%CI 0.68–1.00) for rs1260333; for TT vs. CC, OR is 0.72 (95%CI 0.58–0.88) for rs1260326, and 0.72 (95%CI 0.58–0.89) for rs1260333. Under allele model, for allele T vs. C, the ORs are 0.85 (95%CI 0.76–0.94) and 0.85 (95%CI 0.76–0.94) for rs1260326 and rs1260333, respectively).

**Conclusions:**

Our study confirmed the associations between *GCKR* variants and triglycerides in Chinese children and adults. Triglycerides-increasing alleles of *GCKR* variants reduce insulin and HOMA-IR index, and protect from insulin resistance in children. Our results suggested *GCKR* has an effect on development of insulin resistance in Chinese children.

## Introduction

Type 2 diabetes is a worldwide heath problem. For Chinese, the prevalence of type 2 diabetes is increasing, Yang W. *et al.* reported that the prevalence of total diabetes and prediabetes in China were 9.7% and 15.5%, respectively [1]. Insulin resistance contributes to the pathogenesis of type 2 diabetes, and genetics plays an important role for insulin resistance [2].

Glucokinase regulatory protein (GKRP; gene symbol: *GCKR*) is a rate-limiting factor of glucokinase (GCK), which functions as a key glycolytic enzyme for maintaining glucose homeostasis [3]. Polymorphisms at the *GCKR* gene region were firstly identified to be associated with triglycerides levels by genome wide association studies [4], and the alleles which increasing triglycerides levels were found to lower the glucose, insulin levels and insulin resistance by different association studies[5]–[7]. Rs1260326, non-synonymous variant in *GCKR*, was reported to have inverse effects on triglycerides and glucose levels in French individuals[8]; [9], and other European descent populations [10]. Another common variant rs1260333 which located in downstream of *GCKR* gene region, was reported to be associated with triglycerides in European by Waterworth *et al.* [11]. In Chinese, the association of *GCKR* variants with glucose, insulin, insulin resistance and the risk of type 2 diabetes was inconsistent with Europeans[12]–[16]. Three studies validated the association between *GCKR* SNP and type 2 diabetes mellitus, but Wen J. *et al.* didn’t[12]; [13]; [15]; [16]. Qi Q. *et al.* found *GCKR* SNPs (rs780094 and rs1260326) to be associated with glucose and HOMA-beta function index, but others didn’t[12]–[16]. To better understand the metabolism of glucose-lipid, it is necessary to investigate the relationship of *GCKR* variants with triglycerides and glucose metabolic traits (glucose, insulin, HOMA-IR) for Chinese adults or children.

In the present study, we genotyped two *GCKR* variants rs1260326 and rs1260333 in larger population including two Chinese non-diabetic groups: 1) a children and adolescents population; 2) an adult population. We aimed to investigate the possible effects of rs1260326 and rs1260333 on triglycerides, fasting glucose, insulin and HOMA-IR index, and estimate their effects on the risk of insulin resistance in Chinese non-diabetic children and adults population.

## Methods

### Study Population and Measurements

All subjects are unrelated northern Chinese of the Han ethnicity living in the area of Beijing.

#### 1. Children and adolescents

subjects were recruited from a cross-sectional population-based survey: the BCAMS study [17]. The study population and measurement of anthropometric parameters were collected, which introduced in our previous papers in details[18]; [19].Within this large group, pubertal development was assessed by Tanner stage of breast development (girls) and testicle volume (boys) [20]. The BCAMS study was approved by the Ethics Committee and Institutional Review Board at Capital Institute of Pediatrics (CIP). All participating children and their parents gave written informed consent under protocols provided by the CIP that clearly stated that the blood samples will be used for scientific research purposes including genetic studies. There were 3,518 children and adolescents were recruited and collected venipuncture blood for further tests. In this study, 33 individuals were excluded for FPG≥7.0 mmol/L and/or self-report diabetes during analysis.

#### 2. Adults

subjects were consists of 1,773 non-diabetic participants by OGTT(oral glucose tolerance test, from a community-based health screening program and recruited by local community hospitals of Beijing, which are the cooperation loci of Beijing Hypertension League Institute(BHLI), during 2001–2003. General information was recorded, including age, sex, height, weight, disease history. All subjects gave written informed consent under protocols provided by BHLI that clearly stated that individual data and blood samples will be used for scientific research purposes including genetic studies. The informed consent forms and study purposes were approved by the Ethics Committee and Institutional Review Board of BHLI.

After fasted more than 8 hours or overnight, venous blood from each of these subjects was collected for further tests. All biochemical measurements were performed using commercially available kits. Triglycerides and fasting glucose were measured by enzymatic methods (Roche, Basel, Switzerland)by 7060 chemistry analyzer (Hitachi, Tokyo, Japan), and fasting insulin was measured by enzyme-linked immunosorbent assay. All experiments were carried out according to standard operating procedures. Body mass index (BMI) was calculated according to the formula: weight (kg) divided by squared height (m^2^). Homeostasis model assessment of insulin resistance (HOMA-IR) index was calculated by the formula: (fasting glucose (mmol/L)* fasting insulin (µU/ml)/22.5) [21]. HOMA-IR index was divided into quartiles, and the top quartile was defined as insulin resistance (IR) [22], other three quartiles as controls. The cutoff of HOMA-IR index was 2.79 and 3.38 for insulin resistance diagnosis in our children and adults population, respectively.

### Genome DNA Extraction and Genotyping

Genomic DNA was isolated from peripheral blood white cells using the salt fractionation method. All genotyping were performed by TaqMan probes Allelic Discrimination Assays (Assay number are C___2862880_1_ for rs1260326 and C___8724522_10 for rs1260333) with the GeneAmp 7900 Sequence Detection System (Applied Biosystems, Foster City, CA, USA). Genotyping call rates for all variants were greater than 99%. Duplicate samples were assayed with a concordance rate >99%.

### Statistical Analysis

Quantitative variables were expressed as mean ± standard deviation (SD). The chi square test was used to compare the genotype distribution in the children and adults groups, and perform the Hardy-Weinberg equilibrium test for two variants. Differences between genotypes were tested with multiple linear regression analysis under an additive effect model, for children, adjusted for age, sex, BMI and puberty, and for adults, adjusted for age, sex and BMI. Triglycerides, fasting insulin and HOMR-IR index were square root transformed for approximate normality distribution. Odds ratios (ORs) were calculated using logistic regression analysis model to evaluate the possible associations of *GCKR* variants with insulin resistance. Statistical analyses were performed with SPSS, version 13.0 (SPSS, Inc., Chicago, Illinois), and *p*<0.05 was considered statistically significant.

## Results

The major clinical and metabolic characteristics of children and adults are displayed in Table 1. Both of rs1260326 and rs1260333 were in Hardy-Weinberg equilibrium for each group (*p*>0.05) (Table 2). There is no difference of genotype distribution between children and adults for two variants (*p*>0.05) (Table 2).

**Table 1:** Characteristic of children and adults.

	children	adults
*n*	3,485	1,773
male,%	51	40.2
Age (years)	12.4±3.1	45.1±9.1
BMI (kg/m^2^)	21. 9±4.9	25.1±3.7
triglycerides (mmol/L)	0.89(0.66–1.22)	1.29(0.88–1.94)
TC(mmol/L)	4.09±0.79	5.02±0.90
HDL-C(mmol/L)	1.40±0.32	1.43±0.34
LDL-C(mmol/L)	2.54±0.73	3.38±0.88
fasting blood glucose (mmol/L)	5.07±0.45	5.13±0.55
Fasting blood insulin (µU/ml)	8.35(5.12–12.99)	10.18(7.21–14.60)
HOMA-IR (mmol/L×µU/ml)	1.90(1.13–2.79)	2.28(1.59–3.38)

Data are mean±SD, or median (25%quatile-75%quatile).

HOMA-IR: (fasting blood glucose [mmol/L] * fasting blood insulin [µU/ml])/22.5.

TC: total cholesterol; HDL-C: high density lipoprotein cholesterol; LDL-C: low density lipoprotein cholesterol.

**Table 2:** Genotypes distribution in children and adults population.

		genotype						
SNP	population	CC	CT	TT	MAF	*H-W-P*	chi square	*p* value
rs1260326	children	726	1,711	1,048	0.45	0.57	0.11	0.95
	adults	371	860	538	0.45	0.43		
								
		CC	CT	TT				
rs1260333	children	744	1,682	1,059	0.45	0.11	1	0.61
	adults	397	845	522	0.46	0.12		

*H-W-P*: *p* value for Hardy-Weinberg equilibrium test.

The T alleles of rs1260326 and rs1260333 increased triglycerides for children (for rs1260326, *β* = 0.03, *p = *3.36×10^−9^; for rs1260333, *β* = 0.03, *p = *1.00×10^−9^) and adults (for rs1260326, *β* = 0.06, *p = *8.92×10^−7^; for rs1260333, *β* = 0.06, *p = *4.81×10^−6^) (Table 3). In children, T alleles of rs1260326 and rs1260333 strongly lowered fasting insulin (for rs1260326, *β* = −0.07, *p = *0.003; for rs1260333, *β* = −0.07, *p = *0.003) and HOMA-IR index (for rs1260326, *β* = −0.03, *p = *0.002; for rs1260333, *β* = −0.03, *p = *0.002) (Table 3). No associations were observed with fasting glucose in children (Table 3). For adults, it didn’t reach statistically significance for relationships between the two *GCKR* variants and fasting glucose, insulin and HOMA-IR index (Table 3).

**Table 3:** Clinical and metabolic characteristics of Chinese children and adults stratified according to genotypes of rs1260333 and rs1260326.

SNP	parameters	childrengenotypes			*p* value^a^ (β)	Adults genotypes			*P* value b (β)
		CC	CT	TT		CC	CT	TT	
	*n*	726	1,711	1,048		371	860	538	
rs1260326	BMI (kg/m^2^)	21.9±5.1	22.0±4.9	21.8±4.9	0.60(−0.06) c	25.2±3.8	25.1±3.7	25.1±3.6	0.74 (−0.04)^d^
	Triglycerides(mmol/L)	0.82(0.63–1.16)	0.87(0.65–1.21)	0.94(0.71–1.30)	**3.36×10** ^−**9**^(0.03)	1.20(0.78–1.65)	1.28(0.88–1.96)	1.40(0.95–2.09)	**8.92×10** ^−**7**^ (0.06)
	Fasting glucose(mmol/L)	5.08±0.48	5.07±0.44	5.05±0.45	0.10(−0.02)	5.14±0.56	5.14±0.55	5.09±0.54	0.10 (−0.03)
	Fasting insulin(µU/ml)	8.56(5.02–13.69)	8.40(5.17–12.95)	8.13(5.09–10.28)	**0.003**(−0.07)	10.50(7.45–15.05)	10.18(7.17–14.13)	9.95(7.16–14.19)	0.34 (−0.03)
	HOMA-IR (mmol/L×µU/ml)	1.96(1.11–3.20)	1.91(1.15–2.91)	1.83(1.10–2.75)	**0.002**(−0.03)	2.35(1.65–3.58)	2.30(1.60–3.37)	2.22(1.52–3.27)	0.27 (−0.02)
		**CC**	**CT**	**TT**		**CC**	**CT**	**TT**	
rs1260333	*n*	744	1,682	1,059		397	845	522	
	BMI (kg/m^2^)	21.9±5.1	22.0±4.9	21.8±4.9	0.52 (−0.07)^c^	25.3±4.0	25.1±3.6	25.1±3.6	0.41 (−0.10) d
	Triglycerides(mmol/L)	0.82(0.62–1.15)	0.88(0.66–1.21)	0.94(0.70–1.30)	**1.00×10** ^−**9**^(0.03)	1.22(0.79–1.74)	1.28(0.88–14.28)	1.29(0.94–2.05)	**4.81×10** ^−**6**^ (0.06)
	Fasting glucose(mmol/L)	5.08±0.48	5.07±0.44	5.05±0.45	0.11(−0.02)	5.14±0.56	5.14±0.55	5.09±0.54	0.10 (−0.03)
	Fasting insulin(µU/ml)	8.65(5.02–13.66)	8.44(5.24–12.94)	8.10(2.04–12.30)	**0.003**(−0.07)	10.23(7.46–14.86)	10.23(7.21–14.28)	10.18(7.14–14.61)	0.85 (−0.01)
	HOMA-IR (mmol/L×µU/ml)	1.97(1.11–3.17)	1.92(1.16–2.91)	1.81(1.09–2.75)	**0.002**(−0.03)	2.29(1.66–3.55)	2.31(1.60–3.36)	2.29(1.52–3.30)	0.67 (−0.01)

Data are mean±SD, or median (25%quatile-75%quatile).

a adjusted for age, sex, BMI and puberty for children.

b adjusted for age, sex and BMI for adults.

c adjusted for age, sex and puberty for children.

d adjusted for age, sex for adults.

HOMA-IR = (fasting blood glucose [mmol/L] * fasting blood insulin [µU/ml])/22.5. During linear regression analyzing, triglycerides, fasting insulin and HOMR-IR index were square root transformed for approximate normality distribution.

*p* values were calculated from linear regression assuming an additive model. *P* values<0.05 were shown in bold.

Table 4 showed the association between two *GCKR* variants and insulin resistance in children and adults groups. There were significant associations between two *GCKR* variants and insulin resistance for children (Table 4).

**Table 4:** Logistic regression analysis models of rs1260333 and rs1260326 for insulin resistance (IR).

			children					adults				
SNP			Control	IR	OR(95%CI)	*p1*	*p2*	Control	IR	OR(95%CI)	*p1*	*p2*
rs1260326	genotype	CC	497	228	1			266	105	1		
		CT	1,232	468	0.83(0.69–1.00)	0.051	**0.024**	649	211	0.82(0.63–1.08)	0.17	0.19
		TT	785	258	0.72(0.58–0.88)	**0.002**	**0.002**	411	127	0.78(0.58–1.06)	0.11	0.13
		CT+TT	2,017	726	0.78(0.66–0.94)	**0.008**	**0.004**	1,060	338	0.81(0.62–1.04)	0.10	0.12
	allele	C	2,226	924	1			1,181	421	1		
		T	2,802	984	0.85(0.76–0.94)	**0.002**	**0.002**	1,471	465	0.89(0.76–1.03)	0.13	0.15
rs1260333	genotype	CC	508	233	1			287	110	1		
		CT	1,214	460	0.83(0.68–1.00)	**0.047**	**0.012**	641	204	0.83(0.63–1.09)	0.18	0.35
		TT	792	261	0.72(0.58–0.89)	**0.002**	**0.002**	395	127	0.84(0.62–1.13)	0.25	0.42
		CT+TT	2,006	721	0.78(0.66–0.94)	**0.007**	**0.002**	1,036	331	0.83(0.65–1.07)	0.16	0.33
	allele	C	2,230	926	1			1,215	424	1		
		T	2,798	982	0.85(0.76–0.94)	**0.002**	**0.003**	1,431	458	0.92(0.79–1.07)	0.28	0.45

*p1* unadjusted.

*p2* adjusted for age, sex, BMI and puberty for children, adjusted for age, sex, and BMI for adults.

*P* values<0.05 were shown in bold.

For rs1260326, adjusted for age, sex, BMI and puberty, under co-dominant model, for CT vs. CC, OR (odds ratio) is 0.83(95%CI (confidence interval) 0.69–1.00, *p* = 0.024); for TT vs. CC, OR is 0.72(95%CI 0.58–0.88, *p* = 0.002). Under dominant model, OR is 0.78(95%CI 0.66–0.94, *p* = 0.004) for CT+TT vs. CC. And under allele model, compared with C allele, OR is 0.85(95%CI 0.76–0.94, *p* = 0.002) for T allele. Similar associations were observed for rs1260333 and insulin resistance in children, for CT vs. CC, OR is 0.83 (95%CI 0.68–1.00, *p* = 0.012); for TT vs. CC, OR is 0.72(95%CI 0.58–0.89, *p* = 0.002). For CT+TT vs. CC, OR is 0.78(95%CI 0.66–0.94, *p* = 0.002). For T allele vs. C allele, OR is OR = 0.85(95%CI 0.76–0.94, *p* = 0.003) (Table 4). No significant association between the two *GCKR* variants and insulin resistance was observed for adults (Table 4).

## Discussion

To our knowledge, the present study firstly investigated the effects of *GCKR* variants rs1260326 and rs1260333 on glucose metabolism traits in Chinese population including children and adults. We replicated the association between rs1260326 and rs1260333 and triglycerides in Chinese children and adults, it suggested that the effects of *GCKR* variants rs1260326 and rs1260333 on triglycerides are similar in Chinese and Europeans. Our data firstly find that the triglycerides-increasing alleles of *GCKR* variants rs1260326 and rs1260333 lowered insulin and HOMA-IR, and reduced the risk of insulin resistance in Chinese descent children.

Both of *GCKR* variants rs1260326 and rs1260333, locate in a strong LD in our population (*r* ^2^ = 0.88, 0.89 for children and adults, respectively), and similar to the HapMap data (for CHB, *r* ^2^ = 0.86). For this, it is understandable that the variant rs1260333 has similar effects with rs1260326.

GKRP can activate GCK, which functions as a glucose sensor responsible for glucose phosphorylation in the first step of glycolysis [23]. As a coding variant (P446L), rs1260326 was well described as a potential causal polymorphism. Beer *et al.* reported an *in vitro* experiment that the T allele of rs1260326 had increased the activity of GCK [24]. Higher GCK activity could lead to increase triglycerides but lower glucose [23]. For adults, the association between rs1260326 and glucose, insulin and HOMA-IR were reported by European studies[8]; [9], among Indian Asians, the similar effect was observed by a genome wide association study [25]. In Japanese adults, rs1260326 associated with fasting glucose [26]. For children, effect of rs1260326 with glucose metabolism wasn’t detected, although it associated with triglycerides levels [27]. In our study, the T alleles of *GCKR* increased the triglycerides levels in children and adults didn’t lower glucose levels. This result on glucose was consistent with studies conducted by other Chinese researchers for another *GCKR* variant rs780094[12]; [13], which is in the high LD block with rs1260326 (HapMap CHB *r* ^2^ = 0.82). For the inconsistent results on glucose within Chinese, Europeans and Japanese, we thought it should be due to the different genetic background and life styles with three populations.

We firstly observed that the *GCKR* variants of increasing triglycerides had lower the levels of insulin and HOMA-IR index, and reduce the risk of insulin resistance for Chinese children. There are some limitations in our study. For adults, it displayed similar trends, but didn’t reach the significance. Given that limited sample size of adults, the power wasn’t sufficient (under genetic additive model, to achieve power>80%, OR is 0.85, it needs >800 adult cases with insulin resistance in our study). It is one limitation of our study. Whether or not the *GCKR* variants associate with insulin resistance in Chinese adults, it needs larger-sample population to verify. Another limitation is that there was possible misclassification for diagnosing insulin resistance for children. We only excluded the subjects with FPG> = 7.0 mmol/L and/or self-report diabetes and treatment with anti-diabetic agents, and didn’t considered 2 h postprandial glucose, that maybe introduced misclassification bias.

A transient insulin resistance develops in children during puberty [28]. For children, the insulin resistance emerging during pubertal maturation is accepted as a physiological condition rather than pathologic [29]. Pubertal insulin resistance was accompanied by greater fasting serum insulin concentrations while serum glucose concentrations were unchanged[30]; [31]. Insulin resistance in young adults is often accompanied by a dyslipidemic profile [32]. Prevailing theories for the pathogenesis of insulin resistance focus on lipid-mediated mechanisms [33]. Puberty is a high risk developmental period for type 2 diabetes [34], because the transient physiological status in insulin resistance induces an extra stress on the beta cells in the pancreas(29).Although insulin resistance declines in late puberty [31], the associations between *GCKR* variants and insulin resistance in Chinese children are still important to understand the mechanism of pubertal insulin resistance.

In conclusion, our study confirmed the association between *GCKR* variants and triglycerides levels in Chinese children and adults. The triglycerides-increasing alleles of *GCKR* variants can reduce blood insulin and HOMA-IR index, and the risk of insulin resistance in Chinese children. Our results suggested *GCKR* has an effect on development of insulin resistance in Chinese children. It’s well-known that insulin resistance plays an important role in development of type 2 diabetes, prospective study is needed to estimate whether the variants associate with development of type 2 diabetes in Chinese descent population.
